# Supplementary material for: Are carbon emissions trading and green financial instruments synergistic? -Comprehensive quantitative research based on content analysis
Source: PLoS One. 2024 Mar 7;19(3):e0298601. doi: 10.1371/journal.pone.0298601 (PMC10919601; doi:10.1371/journal.pone.0298601)
Supplement: S1 Data — (ZIP) [file pone.0298601.s001.zip › Data/地级市Z_F工作报告.zip!/╔╜╢1⁄2/╤╠╠¿╩╨/└┤╫╘╣1⁄2╓┌║┼í╛┬φ┐╦╩2╛▌═°í┐_2018.docx]

各位代表：

现在，我代表市人民政府向大会报告工作，请予审议，并请市政协委员和其他列席会议的同志提出意见。

一、2017年工作回顾

2017年是本届政府的开局之年。一年来，全市上下在以习近平同志为核心的党中央坚强领导下，坚持以习近平新时代中国特色社会主义思想为指导，认真贯彻落实省委省政府和市委决策部署，努力推动创新发展、持续发展、领先发展，经济在调控中趋稳，结构在调整中优化，动能在转换中增强，民生在发展中改善，经济社会发展总体平稳、稳中有进。全市实现生产总值7339亿元，增长6.5%；一般公共预算收入600.3亿元，增长6.4%；固定资产投资5594亿元，增长8.5%；社会消费品零售总额3273亿元，增长10%；城镇和农村居民人均可支配收入分别达到41837元和18051元，均增长8%。我市第五次获评全国文明城市、第七次获评全国社会治安综合治理优秀城市、第四次捧得“长安杯”，成为首批国家食品安全示范城市。

(一)着力优化产业结构，发展质量效益进一步提高。工业向集群化、高端化迈进。规模以上工业主营业务收入继续保持全省前列，利润增长11.3%。4个两千亿级、2个千亿级产业和16个百亿级企业稳健发展，医药健康产业总收入增长12%以上，战略性新兴产业产值占比达到22.3%。“关停搬转”化工企业526家，化工产业利润增长90%以上。完成技改投资1602亿元，万元生产总值能耗下降3.4%。工业稳增长促转型成效明显，受到国务院表彰。一批骨干企业勇立潮头、创新发展。万华百万吨乙烯项目启动实施；中集来福士“蓝鲸1号”钻井平台助推我国首次成功试采可燃冰；鲁花集团入选2017年国家品牌计划；绿叶制药成为全国最具研发创新能力医药上市企业10强。服务业向规模化、品质化迈进。金融机构本外币存贷款余额分别达到7933亿元和5099亿元。新增基金和管理机构90家，基金总规模1299亿元。新增7家上市公司，总数达到47家，稳居全省首位。全域旅游示范区创建纵深推进，全年接待游客7000万人次，旅游消费总额940亿元，增长12%。开通集装箱远洋航线，港口货物吞吐量突破4亿吨、集装箱吞吐量270万标箱，烟台港进入全国沿海十大港口行列。机场旅客吞吐量650.3万人次，货邮吞吐量5.1万吨。电子商务交易额增长32.6%。服务业增加值占生产总值比重提高0.8个百分点，达到43.4%。农业向标准化、绿色化迈进。农业“新六产”快速发展，新增农民合作社1500多家、家庭农场700多家。粮食生产保持稳定。新认证“三品一标”农产品129个，创建24个省级以上畜禽标准化养殖示范场。增加1处国家级海洋保护区、5万亩海洋牧场。发展农业节水灌溉面积19万亩，新增水肥一体化面积22万亩。农业机械化水平达到91%。栖霞国家现代农业产业园、龙口省级农业高新技术产业开发区获批建设。我市成为全国农产品冷链流通标准化示范城市。

(二)纵深推进创新改革开放，新旧动能转换步伐进一步加快。积极争取新旧动能转换创新政策和体制机制改革事项，启动编制新旧动能转换重大工程实施规划，筛选建立重点项目库。创新驱动效应持续扩大。我市被确定为济青烟国家科技成果转移转化示范区、全国首批知识产权强市创建市。国家首个在地方设立的中国知识产权保护中心落户烟台。新设国家级核电产业技术创新平台、中德工业设计中心等一批创新载体。上海交大、西南交大分别与我市合作共建信息技术研究院。海军航空大学与我市合作共建军民融合创新示范基地。新增省级以上技术创新中心、企业技术中心、重点实验室27个。新增高新技术企业69家、科技型中小企业291家，高新技术产业产值占比达到42.5%。新增发明专利授权量1238件，12件专利获得中国专利奖。获得3项国家级、15项省级科技奖励。引改革推动效应持续扩大。供给侧结构性改革取得阶段性成果，商品住宅去化周期进入合理区间，企业负债率持续低于全国全省平均水平。新实施国家低碳城市等省级以上改革试点33项，总数达到134项。深化“放管服”改革，市级行政许可事项由260项压缩到96项，政务服务平台“一级建设、三级应用”模式在全省推广。建立服务企业“直通车”制度，168家企业进入省直供电范围，发放信贷周转金9.8亿元，减免企业和社会税费负担300亿元以上。市管企业全面完成公司制改制，营业收入突破1000亿元，增长43.8%，利润增长170.8%。深化财税体制改革，政府财政透明度居全国地级市首位。农村集体产权制度改革试点扎实推进。深化医药卫生体制改革，公立医院全部取消药品加成，药价平均下降27.2%。稳步推进“县管校聘”教师管理体制改革，顺利通过教育信息化区域综合试点验收。国有林场改革任务全面完成。城市信用综合指数稳居全国地级市前列，获得国家城市信用建设创新奖。开放带动效应持续扩大。新引进世界500强企业投资项目19个，实际使用外资145亿元，进出口突破3000亿元。烟台开发区扩区发展，烟台高新区成为国家知识产权示范园区，烟台保税港区进出口总值稳居全国保税港区前列，招远开发区启动体制机制改革试点。国务院批复设立中韩(烟台)产业园。国际友城达到27个。成功举办医药创新与发展国际会议、核电产业链高峰论坛、东北亚海洋经济创新发展示范论坛等活动，推动了产业聚集，扩大了对外影响。

(三)谋划推进重大基础设施，城乡面貌进一步改善。完成城市总体规划实施评估及18项专项规划和11个区片控规，成为全国城市设计试点市。一批事关长远的重大基础设施项目加快布局推进。龙烟铁路建成通车，潍莱高铁开工建设，环渤海潍烟高铁获准提前实施。烟台港西港区一期工程竣工投产。烟台蓬莱国际机场二期工程启动前期工作。蓬栖高速公路建成通车，龙青高速公路加快建设。滨海路开发区至蓬莱段、塔山南路开工建设。新建改造国省干线公路190公里、县乡公路200公里、村级公路800公里。一批事关民生的重点工程启动实施。改造棚户区3万套、老旧小区6.3万户，打通市区断头路23条。启动朝阳街、所城里历史文化街区保护改造，基本完成房屋征收。崆峒岛居民整体外迁。市区完成供水、供暖老旧管网改造457公里。全市新增天然气用户10万户、集中供热295万平方米。建设海绵城市28平方公里。整治违法建筑189万平方米。铁路公路沿线环境整治取得新成效。升级改造污水处理厂9座，生活垃圾资源化利用率达到56%。完成年度农村厕所改造任务。对175座水库进行除险加固。解决了695个村饮用水水质安全问题。为有效缓解旱情调引客水1.1亿立方米。一批县、镇、村积极探索特色发展道路。蓬莱市成为国家级旅游度假区。新创建省级特色小镇7个。美丽乡村覆盖率达到60%。省委省政府出台《关于推进长岛海洋生态保护和持续发展的若干意见》，长岛发展迎来了重大机遇。

(四)集中打好三大攻坚战，和谐稳定局面进一步巩固。打好防范化解重大风险攻坚战，强化金融风险监测预警，稳妥处置金融机构和企业风险，严厉打击非法集资，扎实开展互联网金融风险专项整治，防控金融风险的体制机制进一步完善。深入开展安全生产隐患“大快严”集中行动，生产安全事故起数、死亡人数分别下降23.8%和18.7%。森林防火形势总体平稳。深入推进“三城联创”，完善社会治安防控体系，开展信访积案化解工作，及时妥善处理各类矛盾和问题。全力防控各类风险，圆满完成了十九大安保维稳任务。打好精准脱贫攻坚战，实施112个扶贫产业项目，“扶贫特惠保险”实现应保尽保，208个省定扶贫工作重点村脱贫摘帽，10410名贫困群众稳定脱贫。打好污染防治攻坚战，以中央环保督察为契机，强力整治环境突出问题。自然保护区内违法违规项目清理取得实效。关闭搬迁禁养区内畜禽养殖场2525个。所有河流全部实行河长制，境内22条黑臭水体全部完成整治。完成造林及修复14万亩，治理水土流失27.6万亩。扎实推进气代煤、电代煤，淘汰燃煤小锅炉3198台，压减煤炭消费92万吨。新增新能源公交车1100辆，成为全省绿色交通示范市。空气质量四项主要污染物浓度年均值达到国家二级标准。

(五)大力发展社会民生事业，群众生活水平进一步提升。民生支出548.2亿元，占财政支出比重达到77.4%。新增城镇就业14.5万人，城镇登记失业率控制在3.26%。完成退休人员基本养老金调整和全民参保登记，企业职工养老金实现“十三连增”。免费为所有家庭投保自然灾害意外伤害保险和家庭房屋财产损失保险。职工大病保险制度全面实施，居民大病保险年最高报销额由30万元提高到50万元，跨省异地就医实现即时结算。启动职工长期护理保险试点。医联体建设覆盖所有公立医院和乡镇卫生院。医养结合示范县达到8个，居全省首位。县均城市和农村低保标准分别提高到每人每月580元和374元。发放各类救助金5.3亿元，救助困难群众20.3万人次。解决“大班额”问题取得阶段性成效，“全面改薄”工作扎实推进，新建改建学校78所、校舍55万平方米，开工建设幼儿园73所，新增幼儿学位1.1万个，“国家级学校安全教育示范区”考核位列地市级首位。全民健身运动蓬勃发展，烟台籍运动员在第十三届全运会上赢得44枚奖牌。开展文化惠民演出8万多场、品牌阅读活动370多场。“善行义举四德榜”实现村居全覆盖。实现一村(社区)一法律顾问。有序推进村(居)委会换届选举工作。双拥共建、国防动员、民兵预备役、人民防空工作顺利开展。援藏援疆和扶贫协作工作取得实效。人口计生工作保持先进水平。老龄、妇女、儿童、残疾人、红十字事业健康发展，统计、史志、档案、仲裁、机关事务、新闻出版、广播电视、民族宗教、国家安全和气象、地震、打私等工作取得新成绩。

(六)坚持从严治党、从严治政，政府自身建设进一步加强。深入学习宣传贯彻党的十九大精神，推进“两学一做”学习教育常态化制度化，树牢“四个意识”，坚定“四个自信”，旗帜鲜明讲政治，坚决维护以习近平同志为核心的党中央权威和集中统一领导。认真执行市人大决议决定，主动与市政协协商工作，办理市人大代表建议219件、政协提案448件，提请市人大常委会审议了安全生产监督管理、城市供水2项地方性法规草案。深入推进依法行政，修订了政府工作规则，健全了法律顾问制度，规范了重大行政决策程序。全面推进政务公开，编制公开了市县乡公共服务清单。启动政务信息系统和政务服务热线整合工作。全年受理市长公开电话、市长信箱等反映事项4万多件，办结率达到99.2%。建立10个领导小组工作推进机制，集中精力抓重点、补短板、强弱项，有力推动了重点工作落地见效。强化行政监察和审计监督，持之以恒改进作风，坚持不懈反腐倡廉，政务生态环境持续改善。

各位代表，过去一年的成绩来之不易。这是以习近平同志为核心的党中央坚强领导的结果，是省委省政府正确领导、关心支持的结果，是市委团结带领全市人民努力奋斗的结果，是市人大、市政协和社会各界监督支持的结果。在此，我代表市人民政府，向全市人民、各界人士，向驻烟部队、武警官兵和公安干警，向中央省属驻烟单位，向老领导、老同志，向所有关心支持烟台发展的海内外朋友们，表示衷心的感谢并致以崇高的敬意！

各位代表，在肯定成绩的同时，我们也清醒地看到，发展中还存在不少困难和挑战。在经济由高速增长转向高质量发展的阶段，还面临着产业结构不优、创新动力不足等问题，新动能的增量尚不足以对冲旧动能的减量，经济增速趋缓，部分指标低于预期目标。一些企业生产经营困难，中小微企业融资难、融资贵的问题仍然突出。外贸结构不够优化，利用外资质量不够高，缺少具有支撑力的大项目好项目。安全生产、环境保护等领域还存在薄弱环节。城乡基础设施建设存在短板，公共服务均等化水平有待提高。就业、教育、医疗、居住、养老等民生工作需要加强和改善。政府工作人员的法治思维、专业素养需要进一步提升，不正之风和腐败问题时有发生。对上述问题，我们一定高度重视，采取有效措施，认真加以解决。

二、2018年政府工作指导思想和奋斗目标

2018年是贯彻党的十九大精神的开局之年，是改革开放40周年，是决胜全面建成小康社会、实施“十三五”规划承上启下的关键一年。今年的政府工作，要深入贯彻党的十九大精神，以习近平新时代中国特色社会主义思想为指导，认真贯彻落实习近平总书记视察山东重要讲话和重要指示批示精神，坚持稳中求进工作总基调，坚持新发展理念，按照高质量发展的要求，统筹推进“五位一体”总体布局和协调推进“四个全面”战略布局，坚持“一个定位、四个着力、一个加快”发展思路，以供给侧结构性改革为主线，以实施新旧动能转换重大工程为统领，以发展壮大实体经济为重点，加快建设现代化经济体系，纵深推进改革开放创新，着力保障和改善民生，全力维护社会和谐稳定，在全面建成小康社会进程中走在前列。

今年全市经济社会发展主要预期目标是：生产总值增长7%以上，一般公共预算收入增长6.5%左右，固定资产投资增长8%左右，社会消费品零售总额增长10%左右，外贸进出口、实际使用外资保持平稳增长，城镇和农村居民人均可支配收入分别增长7.5%和8%左右，完成省下达的节能减排降碳约束性指标。

各位代表，我市正处于新旧动能转换的关键时期，实现上述目标，需要全市上下锐意进取、奋力拼搏、埋头苦干。我们必须牢牢把握习近平新时代中国特色社会主义思想的精髓要义，坚定不移地用以武装头脑、指导实践、推动工作。我们必须牢牢把握高质量发展要求，坚持质量第一、效益优先，使创新成为第一动力、协调成为内生特点、绿色成为普遍形态、开放成为必由之路、共享成为根本目的。我们必须牢牢把握稳中求进的工作总基调，把发展的着力点放在实体经济上，保持战略定力，实现经济平稳健康发展和社会和谐稳定。我们必须牢牢把握走在全省前列的目标定位，着力打造先进制造业强市、现代物流中心、健康养生胜地、文化旅游名城，加快建设创新开放包容、宜居宜业宜游的现代化国际滨海城市。我们必须牢牢把握事关长远的重要工作，坚持发展思路不变，坚持工作力度不减，坚持10个领导小组工作推进机制，进一步聚焦重点、集中发力、攻坚突破，持之以恒抓落实，久久为功求实效。我们必须牢牢把握以人民为中心的发展思想，抓住群众最关心最直接最现实的利益问题，既尽力而为、又量力而行，一件事情接着一件事情办，一年接着一年干，让广大群众的获得感、幸福感、安全感更加充实、更有保障、更可持续。

各位代表，目标引领方向，目标凝聚力量。我们坚信，在中共烟台市委的坚强领导下，依靠700多万烟台人民的智慧和力量，一定能够战胜前进道路上的各种艰难险阻，一定能够创造出无愧于时代的发展成就，一定能够开创烟台更加美好的未来！

三、2018年主要任务

做好今年的政府工作，要深入贯彻党的十九大精神，按照中央、省经济工作会议部署，统筹推进稳增长、促改革、调结构、惠民生、防风险各项工作，坚决打好三大攻坚战，聚焦十项重点工作持续加力，努力保持经济社会平稳健康发展。

坚决打好三大攻坚战：一是坚决打好防范化解重大风险攻坚战。加强金融风险防控。完善金融管理协作机制，促进法人金融机构和金融组织规范健康发展。着力防范化解担保圈和企业流动性风险，扎实推进互联网金融风险整治，依法严厉打击恶意逃废金融债务等违法行为，坚决遏制投资理财等领域非法集资行为，守住不发生系统性金融风险底线。加强安全生产管理。开展“企业安全生产责任落实年”活动，健全风险管控和隐患排查治理体系，加大危化品、道路交通、建筑施工、海上运输、矿山、消防等重点领域综合整治力度，坚决防范各类安全生产事故发生。加强食品药品安全监管。提高食品药品抽检率，推动4000家餐饮服务单位实现“明厨亮灶”，构建从田间到餐桌的全过程食品安全监管体系，保障群众饮食用药安全。努力维护社会稳定。深化“三城联创”，强化公安基层基础工作，完善社会治安防控体系，加强反恐怖、反邪教、反走私工作，依法打击和惩治黄赌毒黑拐骗等违法犯罪活动。完善社区治理体系，发挥社会组织作用，推动社会治理重心向基层下移。做好信访、仲裁、行政复议工作，着力化解社会矛盾。强化应急处置体系建设，做好气象服务、防震减灾、防汛抗旱、森林防火、海上搜救等工作，提升防灾减灾救灾能力。二是坚决打好精准脱贫攻坚战。动员全市各级高度重视起来，动员全社会积极行动起来，尽锐出战，精准施策，确保全市人民一个不少、一户不落奔小康。集中攻坚30个扶贫重点镇、200个扶贫重点村，对6.8万建档立卡贫困人口脱贫不脱政策，巩固提升脱贫成果；对2215名新识别和返贫人口开展精准帮扶，年内实现稳定脱贫。推进扶贫开发与农村低保政策相衔接，全面落实医疗、教育、住房安全保障政策，实施扶贫特惠保险，提升贫困人口“两不愁三保障”质量。大力推行孝善养老、公益专岗扶贫，发挥社会救助兜底保障作用，解决特殊贫困群众生活困难。加大涉农资金整合力度，加强扶贫资金监管。坚持扶贫与扶志、扶智相结合，激发贫困人口内生脱贫动力，确保脱真贫、真脱贫。三是坚决打好污染防治攻坚战。抓好中央环保督察反馈意见整改，做到立行立改、彻底整改。做好全国污染源普查工作，开展县市区环境保护督察，坚决惩处破坏生态环境行为。扎实推进大气污染防治行动，深入治理燃煤、汽车尾气、扬尘等污染，推广清洁煤炭45万吨、节能环保炉具6万台，继续开展气代煤、电代煤行动，压减煤炭消费量，空气质量稳定达到国家二级标准并持续改善。扎实推进水污染防治行动，强化涉水企业污染整治，启动4个污水处理厂扩建项目，完成94公里雨污分流改造，建设全国水生态文明城市。严格落实河长制，加强河道及水源地环境整治。扎实推进净土行动，实施土壤改良修复、农药残留治理等7项工程，加强农业面源污染防治。落实最严格的耕地保护制度和节约集约用地制度。启动实施国土绿化提升三年攻坚行动，深入开展森林城市和森林镇村创建活动，加强“山水林田湖草”生态保护，完成造林和生态修复11万亩、森林抚育20万亩、湿地保护与恢复8万亩，治理水土流失面积180平方公里。完善自然保护区发展规划，排查清理违法违规建设项目及开发活动。打好三大攻坚战是中央和省作出的重大战略部署，必须作为决胜全面小康的重要任务，作为推动高质量发展的重要举措，作为实现稳中求进的重要内容，时刻放在心上，牢牢抓在手中，确保取得阶段性胜利。

聚焦十项重点工作持续加力：

(一)聚焦创新驱动发展，在实施新旧动能转换重大工程上持续加力。坚持以新技术、新产业、新业态、新模式，促进产业智慧化、智慧产业化、跨界融合化、品牌高端化。设立新旧动能转换基金，抓好192个市级重点项目，在山东新旧动能转换综合试验区建设中率先突破、走在前列。探索实施创新政策。落实《山东新旧动能转换综合试验区建设总体方案》，突出产业体系、经济体制、创新驱动、需求拉动四个着力点，在打造海洋生物医药产业集群、开展基金管理服务专项改革创新、创建国家服务外包示范城市等方面先行先试，推动经济发展质量变革、效率变革和动力变革。着力打造创新载体。推进山东半岛国家自主创新示范区、济青烟科技成果转移转化示范区等重大创新载体建设，发挥国家级核电产业技术平台、海工装备研发平台、新药研发平台以及技术创新中心、企业技术中心、重点实验室作用。支持高校设立与我市优势产业相契合的专业，支持“校所企”共建协同创新平台，支持国内外创新成果来烟转移转化，推动“校所城产”融合发展。高标准创建国家知识产权强市，发挥中国(烟台)知识产权保护中心功能，为企业和高校院所提供便利化专利服务。建设国家专利导航产业发展试验区、专利技术转移转化产业园。积极培育创新主体。支持企业创新发展，推动规模以上企业建立研发机构，开展市级重大科技项目攻关，培育一批科技型中小企业，新增高新技术企业80家以上。引进培养创新人才。大力实施“双百计划”，加强顶尖人才(团队)引进和“金蓝领”培训，发放第二批烟台“优才卡”，三年筹集1万套人才公寓，引进培养一批具有国际水平的科技领军人才和高水平创新团队，让各类人才创造活力充分迸发、聪明才智充分涌流。

(二)聚焦主导产业培育，在建设先进制造业强市上持续加力。积极构建实体经济、科技创新、现代金融、人力资源协同发展的产业体系，重点培育“7+N”主导产业集群，促进产业迈向中高端水平。围绕培强做大电子信息、食品加工、有色及贵金属、高端化工新材料、装备制造、汽车制造、医药健康等7个优势产业，逐一研究战略规划、突破方向、扶持重点和服务平台。千方百计推动开发区新能源汽车、南山乙烷综合利用、烟台港西港区LNG、海上清洁能源综合供给平台等重大产业项目落地开工，全力推进万华百万吨乙烯、上汽通用东岳动力总成、国际生物医药与健康产业创新中心、中集来福士深海平台试航基地等重大产业项目加快建设，着力打造高端石化基地、国家核电研发和产业化基地、新能源汽车基地、军民融合高技术产业基地、海工装备及高技术船舶创新中心、国际生命科学创新示范区。谋划布局人工智能、虚拟现实、共享经济等一批前沿产业。积极发展数字经济，推动互联网、大数据、云计算等与实体经济深度融合。深化与腾讯云合作，推动传统优势产业数字化转型，构建新型数字经济共同体。推进供应链创新与应用，设立供应链创新基金，积极发展供应链金融，引进和培育供应链示范企业，建立供应链上下游企业合作共赢的协同发展机制。大力发展智能制造、绿色制造、服务型制造，促进信息技术向制造业各环节渗透，推动生产方式向柔性、智能、精细转变。开展消费品工业“三品”战略示范试点，创建全国质量强市示范城市。培强做大50户龙头带动型企业和50户创新引领型企业，培育更多行业单项冠军。大力弘扬企业家精神和工匠精神，尊重企业家，关心企业家，注重对老一代企业家的培训和新生代企业家的培养，完善企业“直通车”制度，构建“亲”“清”新型政商关系，让广大企业扎根烟台沃土，放眼世界谋发展，回报烟台做贡献。

(三)聚焦提质增效，在发展现代服务业上持续加力。推动生产性服务业向专业化和价值链高端延伸。大力发展冷链物流、智能物流、第三方物流等现代物流业，建设临港产业区、济铁烟台物流园、邮政陆运中心等载体。加快建设中德工业设计中心。争取国家新型优质服务业集群建设试点。促进“快递+电商”深度融合，打造特色电商小镇。大力发展现代金融业，依托我市经济体量大、实体经济强的优势，引进银行、保险、证券等金融机构，支持现有金融机构提升管理层级，鼓励法人金融机构做优做强，增强金融服务实体经济能力。支持企业上市和挂牌，提高直接融资能力。加快区域性基金管理中心建设，新增基金350亿元。鼓励发展商业保理、工程咨询、科技服务等各类生产性服务业。推动生活性服务业向便利化和高品质转变。支持商贸业打造质量和信誉品牌，创新个性化、体验式消费模式，发展商业综合体、专业市场、特色街区和社区便利店等商业形态。积极发展医养结合产业，新建医养结合服务机构20家以上，新增医养结合床位1万张以上。推动文化、体育、信息、家政、娱乐等生活性服务业规范化、智慧化、连锁化、品牌化发展，做大规模、做强优势、做足特色。

(四)聚焦乡村振兴战略，在推进农业农村现代化上持续加力。编制实施乡村振兴战略规划，建立健全城乡融合发展体制机制和政策体系。围绕产业兴旺，支持发展现代种植业，依托农业龙头企业，培育果品、水产品、畜禽、蔬菜、茶叶等特色农产品名优品牌，推进苹果产业转型升级，加快从增产导向转向提质导向。深入实施优质粮食工程，守住粮食安全底线。高标准建设国家级现代农业产业园，整建制创建国家农产品质量安全市。建设林果生产机械化示范基地。争创省级以上畜禽标准化养殖示范场25个。壮大新型农业经营主体，健全农业社会化服务体系，支持企业与合作社、农户共建标准化生产基地。新认证“三品一标”100个以上，新建省级农业标准化基地20个，发展粮食高产创建核心区2万亩、节水灌溉16万亩、水肥一体化26万亩。围绕生态宜居，开展农村人居环境三年整治行动，改善农村基础设施。推进“四好农村路”建设，改造农村公路400公里。推动“厕所革命”，基本完成农村无害化卫生厕所改造任务。解决12万农村群众饮水安全问题。实施美丽乡村建设“双百工程”，“美丽乡村”覆盖率达到65%。围绕乡风文明，开展移风易俗行动，培育崇德向善的文明乡风、良好家风、淳朴民风，不断提高乡村社会文明程度。围绕治理有效，加强基层基础工作，全面完成农村社区服务中心提升工程，健全自治、法治、德治相结合的乡村治理体系。培养造就一支懂农业、爱农村、爱农民的“三农”工作队伍。围绕生活富裕，大力发展特色经济，创新发展农业“新六产”，积极培育农村电商、休闲农业、乡村旅游等新业态，拓宽农民增收渠道，提高农民收入水平。实施150个库区扶持项目。培育一批产业聚集型、创新创业型、文化传承型、强农惠农型特色小镇。

(五)聚焦重要领域和关键环节，在推进各类改革上持续加力。深化供给侧结构性改革，扩大优质增量供给。完善国有企业法人治理结构，推进混合所有制改革，推动经营性国有资产统一监管，促进国有资本做强做优做大。深化财税体制改革，完善全口径政府预算管理制度。深化农村土地制度改革，全面推开农村集体产权制度改革，完善承包地“三权”分置制度。深入推进综合行政执法体制改革，促进规范公正文明执法。完成环保机构监测监察执法垂直管理改革。深化医药卫生体制改革，全面推行药品和医用耗材集中带量采购。扎实推进从事生产经营活动事业单位转企改制。持续优化营商环境，全面落实税费减免政策，继续清理涉企收费，降低实体经济成本，落实促进非公有制经济发展政策，支持各类市场主体健康发展。加强诚信体系建设，健全联合惩戒机制，创建国家社会信用体系示范城市。

(六)聚焦营造开放发展新优势，在推动全面对外开放上持续加力。以改革开放40周年为契机，研究谋划新一轮高水平开放政策措施，策划举办系列宣传推介活动，深度融入“一带一路”建设，打造海上合作战略支点，形成全面开放新格局。在招商引资上精准发力。坚持“以项目看发展论英雄”，积极对接世界500强、国内500强、央企和行业领军企业，加大精准招商、专业招商、以商招商力度，新引进世界500强企业投资项目15个以上。围绕制造业“7+N”主导产业集群，开展全产业链招商，千方百计引进具有支撑力和带动力的大项目好项目。鼓励外资投向现代服务业，参与基础设施和公共服务领域投资运营。在优化贸易结构上狠下功夫。实施品牌出口增长行动，扶持骨干外贸企业创新发展，建设加工贸易转型示范基地，自主品牌产品出口占比提高到15%以上。创新发展服务贸易，培育跨境电商、外贸综合服务、市场采购贸易等新业态。有效引导对外投资，带动产品、技术、标准出口。在园区发展上集中突破。烟台开发区要更好发挥开放龙头作用，烟台高新区要探索推行大部门制改革，烟台保税港区要进一步发挥特殊政策功能、培育特色产业平台，东部新区要明确产业定位、强化产业招商，招远开发区和省级开发区要开展行政管理、人事制度、投资服务等体制机制创新。制定中韩(烟台)产业园建设方案，打造中韩地方经济合作和高端产业合作新高地。加快复制推广自贸区政策，提高贸易便利化水平。

(七)聚焦城市提升工程，在推进城市化上持续加力。坚持以人为本、生态优先、规划引领、陆海统筹、品质至上、科学管理，走港产城融合、城乡一体化发展道路。优化城市布局，促进城区融合，提高中心城市首位度。启动新一轮城市总体规划编制，加快编制专项规划、控制性规划，高水平做好城市设计，深入推进“多规合一”，构建全域规划体系。实施“两铁两港两路一水”重大基础设施工程，开工建设环渤海潍烟高铁，计划2021年建成通车。开工建设大莱龙铁路扩能改造工程，计划2020年建成通车。开工建设烟台蓬莱国际机场二期工程，计划2021年建成启用。开工建设烟台港西港区原油码头二期和通用泊位工程，计划2020年建成投用。开工建设文莱高速公路，建成龙青高速公路，全力争取国道G228烟台城区段改线、高速G18烟台段外移项目尽早实施。开展全域治水三年攻坚行动，统筹实施河库水系连通等150个重点水利项目。增强全民节水意识，大力实施市区再生水利用工程，两年内实现应用尽用。配合推进黄水东调应急二期工程。到2020年新增供水能力4.2亿立方米，基本满足特枯年份用水需求。推进“一湾两街区”保护改造，坚持世界眼光、国际标准、烟台特色，统筹推动芝罘湾港区搬迁、规划、招商和建设，精心实施朝阳街、所城里保护修缮，打造传承开埠历史、彰显文化底蕴、富有时代气息的城市经典。加大“三改一通”攻坚力度，改造棚户区10万户以上，改造老旧小区240个，改造背街小巷307条，打通市区断头路40条。推进塔山南路、塔山北路等30条城市道路工程，滨海路开发区至蓬莱段建成通车。精细化管理城市，突出滨海沿线、城市主干道、出入口等重点区域，深入推进城市亮化、美化、绿化、净化。实施智慧交通、智慧环卫、智慧市政等工程。新增和优化调整公交线路35条，新建改造公交站亭115座，创建国家公交都市。引进市区第二气源，新增天然气用户2万户。建设海绵城市28平方公里。新增集中供热200万平方米，集中供热率达到79%。启动市区生活垃圾分类试点，建设生活垃圾焚烧发电等配套工程。统筹城乡发展，开展国家和省级新型城镇化综合试点，促进城乡基础设施、要素配置、基本公共服务等均衡布局，增强对农业转移人口的吸引力和承载力。

(八)聚焦陆海统筹，在建设海洋强市上持续加力。围绕做大海洋产业，加快建设国家海洋经济创新示范城市，设立海洋产业发展基金，培育壮大海工装备、海洋生物医药等特色产业，建设8个海上多功能综合平台，创建国家海洋经济发展示范区。加快“海上粮仓”建设，实现由浅海向深海养殖发展，打造国家级海洋牧场示范区。围绕做强海洋科技，加快推进国家级海洋高技术产业基地建设，支持涉海机构和企业开展重大科技创新，建设8处省级以上海洋工程技术协同创新中心，打造海洋科技产业聚集示范区。培育东方海洋等10家渔业种业龙头企业，做强海参、扇贝等种业基地，打造“水产种业硅谷”。围绕做优海洋生态，推进国家级海洋生态文明示范区建设，实施蓝色海湾、生态岛礁等修复工程，清理规范近海养殖，确保近岸海域海洋功能区水质达标率100%。推进长岛海洋生态保护和持续发展，创建国家生态文明试验区、旅游度假区、军民融合发展示范区。

(九)聚焦文化旅游融合发展，在打造文化旅游名城上持续加力。充分发挥国家历史文化名城和国家公共文化服务体系示范区优势，坚持以文化为灵魂，以旅游为载体，做活文化旅游融合发展文章。培育和践行社会主义核心价值观，弘扬和传承中华优秀传统文化，振兴京剧码头，提升胶东红色文化、开埠文化、葡萄酒文化、仙道文化等特色文化影响力。开展丰富多彩的群众性精神文明创建活动，巩固提升全国文明城市建设成果。完善公共文化服务体系，推进书香烟台建设。举办第二届文化惠民消费季，组织开展400场惠民巡演。高水平建设国家全域旅游示范区，推进海阳、蓬莱国家级旅游度假区建设，高标准打造从昆嵛山至长岛滨海黄金旅游线，加快实施张裕工业文化旅游、莱阳丁字湾恒大旅游综合体等20个重点文旅项目。讲好烟台故事，传播烟台文化，让仙境海岸、魅力烟台享誉海内外。

(十)聚焦补短板强弱项，在保障和改善民生上持续加力。加大民生投入，增加基本公共服务供给，让改革发展成果更多更公平惠及全市人民。提高就业质量和收入水平，实施就业优先战略和积极就业政策，支持高校毕业生留烟就业创业，城镇新增就业13万人以上，城镇登记失业率控制在3.5%以内。健全政府、工会、企业共同参与的协商协调机制，构建和谐劳动关系。完善社会保障体系，实施全民参保计划，提高养老、医疗等社会保障水平，全面推开职工长期护理保险制度，开展居民长期护理保险试点。逐步建立多主体供给、多渠道保障、租购并举的住房制度，为1.4万户住房保障家庭发放租赁补贴。提高城乡低保标准，做好特困人员救助供养工作。落实各项福利保障制度和优抚安置政策，发展残疾人事业和现代慈善公益事业。健全农村留守儿童和妇女、老年人关爱服务体系。优先发展教育事业，均衡发展城乡义务教育，完成“全面改薄”任务，建立消除城镇普通中小学“大班额”长效机制，着力解决中小学生课外负担重、“择校热”等突出问题。启动第三期学前教育行动计划，改扩建一批普惠性幼儿园，两年内新增2.6万个幼儿学位。实施高中阶段教育普及攻坚计划和职业教育内涵建设工程，支持驻烟高校发展，办好特殊教育和网络教育。建设国家健康城市，完善公共卫生服务体系，强化医疗、医保、医药“三医联动”，加快医学中心及专科医院建设，健全分级诊疗体系，大力培养全科医生，提高家庭医生签约服务水平，推进中医药传承发展。完成10个县市区妇幼保健机构标准化工程建设。统筹发展群众体育、竞技体育和体育产业，打造城市社区“十分钟健身圈”。加强援藏援疆和扶贫协作工作。

加快市县档案馆建设，做好史志、民族宗教、红十字等工作。加强国防教育、国防动员、民兵预备役、人民防空工作，争创新一届双拥模范城，巩固军政军民团结局面。

四、加强政府自身建设

新时代、新征程，政府工作必须要有新气象、新作为。全市政府系统要深入学习贯彻党的十九大精神，把牢政治方向，转变政府职能，提高行政效能，强化责任担当，建设人民满意的服务型政府。

一是始终坚持旗帜鲜明讲政治，保持政治定力、思想定力。牢固树立“四个意识”，坚定“四个自信”，坚决维护党中央权威和集中统一领导，自觉在政治立场、政治方向、政治原则、政治道路上同以习近平同志为核心的党中央保持高度一致，做到党中央提倡的坚决响应、党中央决定的坚决执行、党中央禁止的坚决不做。认真开展“不忘初心、牢记使命”主题教育，深刻领会和把握习近平新时代中国特色社会主义思想，全面贯彻党的基本理论、基本路线、基本方略。不折不扣贯彻落实省委省政府和市委决策部署，确保令行禁止、政令畅通。

二是始终坚持以人民为中心，为人民干事、让人民满意。践行以人民为中心的发展思想，把人民对美好生活的向往作为奋斗目标，把群众的安危冷暖时刻放在心上，把群众路线贯穿到政府工作全过程，用心用力解决好群众普遍关心的生态保护、城乡环境、交通拥堵等各类民生问题。深化“放管服”改革，探索组建行政审批局，实行“一枚印章管审批”。加强政务服务标准化建设，继续推进“零跑腿”“只跑一次”改革，全力打造审批事项少、办事效率高、服务质量优的政务环境。全面推行代办制，优化一窗受理、并联审批、容缺审批等制度，强化事中事后监管。深入开展“减证便民”专项行动，实行证照统一免费寄送。开工建设市民中心。整合12345政务服务热线，更好地解决群众的操心事、烦心事。完成政务信息系统整合，实现政务信息资源跨部门、跨地域、跨层级共享。

三是始终坚持依法行政，建设法治政府、阳光政府。遵守宪法和法律，坚持用法治思维和法治方式加快改革、推动发展、化解矛盾。深入实施“七五”普法，巩固“法德共进”成果。自觉接受市人大及其常委会的法律和工作监督，认真执行有关决议决定，主动报告政府工作。加强同市政协的民主协商，接受民主监督，认真听取各民主党派、工商联和人民团体的意见建议。积极开展地方立法工作，充分发挥地方立法的引领和推动作用。坚持科学民主依法决策，严格执行重大行政决策法定程序。深入推进政务公开，让公众更多参与政策制定、执行和监督，促进政府有效施政。

四是始终坚持干字当头，解放思想创新干、勇于担当务实干。“幸福都是奋斗出来的”，我们必须拿出改革开放再出发的决心和勇气，坚决破除自满自足思想，认清差距，找准短板，对标先进，加压奋进；坚决破除路径依赖、方法依赖，善谋新招、善用新法；着力解决本领恐慌问题，增强“八种本领”，建设高素质专业化干部队伍；着力解决“推绕拖”“庸懒散”问题，提振干事创业精气神。大力倡树“创新、一流、务实、高效、担当”的工作作风，坚持目标导向、问题导向，健全完善激励约束机制，旗帜鲜明地为敢于担当、踏实做事、不谋私利的干部撑腰鼓劲。

五是始终坚持全面从严治党，筑牢底线、不越红线。把纪律和规矩挺在前面，强化政治纪律和组织纪律，带动廉政纪律、群众纪律、工作纪律、生活纪律严起来。深入落实中央八项规定实施细则精神和省委实施办法、市委实施意见，持之以恒整治“四风”。运用好监督执纪“四种形态”，抓早抓小，防微杜渐。支持监察体制改革，自觉接受监察委员会监督，强化审计监督，坚决查处损害群众利益的不正之风和腐败问题，打造忠诚、干净、担当的干部队伍，树立为民、务实、清廉的政府形象。

各位代表，习近平总书记强调指出，“中华民族伟大复兴，绝不是轻轻松松、敲锣打鼓就能实现的。全党必须准备付出更为艰巨、更为艰苦的努力”。站在新的历史方位，我们重任在肩、责无旁贷，必须做坚定者、奋进者、搏击者，决不做犹豫者、懈怠者、畏难者，以时不我待、只争朝夕的精神，奋力走好新时代的长征路。让我们更加紧密地团结在以习近平同志为核心的党中央周围，在省委省政府和市委的坚强领导下，高举旗帜、坚定信念，不忘初心、牢记使命，干在实处、走在前列，全力推动烟台创新发展、持续发展、领先发展，为建设现代化国际滨海城市而努力奋斗！
